# A Novel *TP63* Missense Mutation in the Sumoylation Motif Causes Isolated Split‐Hand/Foot Malformation 4: A Pedigree Report and Literature Review

**DOI:** 10.1002/mgg3.70140

**Published:** 2025-09-18

**Authors:** Wei Yang, Jian Zhou, Nuo Si, Xue Zhang

**Affiliations:** ^1^ Department of Medical Genetics Capital Institute of Pediatrics Beijing China; ^2^ McKusick‐Zhang Center for Genetic Medicine, State Key Laboratory for Complex Severe and Rare Diseases Institute of Basic Medical Sciences Chinese Academy of Medical Sciences, School of Basic Medicine Peking Union Medical College Beijing China

**Keywords:** EEC3, split‐hand‐foot malformation 4, SHFM4, sumoylation, *TP63*

## Abstract

**Background:**

Heterozygous *TP63* mutations cause a spectrum of disorders including split‐hand/foot malformation 4 (SHFM4) and ectrodactyly‐ectodermal dysplasia‐cleft lip/palate syndrome 3 (EEC3). While some SHFM4 mutations concurrently induce EEC3‐like phenotypes (designated SHFM4/EEC3 mutations), their prevalence and distribution—particularly those near the p63 C‐terminus—remain poorly characterized.

**Method:**

A multigenerational Chinese family with an isolated form of SHFM was investigated. Genetic analysis included real‐time quantitative PCR and Sanger sequencing. Disease mutation databases and literature were systematically reviewed to identify all reported *TP63* mutations causing isolated SHFM4 and to classify these mutations by clinical phenotypes.

**Results:**

We identified a novel likely pathogenic variant (NM_003722.5: c.2032G>C, p.E678Q) within a sumoylation motif near the C‐terminus of p63. Analysis of 72 families (182 carriers) revealed 28 SHFM4‐causing *TP63* mutations, comprising 12 dual‐phenotype SHFM4/EEC3 mutations and 16 isolated SHFM4‐only mutations. Certain clinical traits of SHFM4 mutations and distribution characteristics for SHFM4‐only mutations were observed.

**Conclusions:**

This study expands the SHFM4 mutation spectrum, demonstrating significant overlap between SHFM4 mutations and EEC3 mutations. The p.E678Q represents the most reliable SHFM4‐only mutation near the protein C‐terminus. These findings will improve molecular classification and genetic counseling for *TP63*‐related disorders.

## Graphical abstract

The expanded SHFM4 mutation spectrum encompasses 28 *TP63* variants, including 12 dual‐phenotype (SHFM4/EEC3) mutations and 16 isolated SHFM4‐only mutations. The novel p.E678Q missense variant in the sumoylation motif is the most reliable SHFM4‐only mutation identified at the C‐terminus to date.

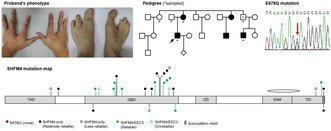

**Abbreviations**
AEC: ankyloblepharon‐ectodermal defects‐cleft lip/palate syndrome
DBD: DNA binding domain
EEC3: ectrodactyly, ectodermal dysplasia, and cleft lip/palate syndrome 3
HGMD: human disease mutation database
PTM: post‐translational modification
qPCR: quantitative polymerase chain reaction
RHS: Rapp–Hodgkin syndrome
SAM: sterile alpha motif domain
SHFM: split‐hand/foot malformation
SUMO: small ubiquitin‐like modifier
TAD: transactivation domain
TID: transactivation inhibitory domain
TP63: tumor protein p63
VUS: variants of uncertain significance

## 1 Introduction

Split‐hand/foot malformation (SHFM) is a congenital limb malformation typically affecting the central rays of the autopod, characterized by median clefts of hands and feet, monodactyly, syndactyly, and aplasia/hypoplasia of the remaining phalanges, metacarpals, and metatarsals. SHFM can occur as an isolated condition or in association with other developmental defects (Online Mendelian Inheritance in Man 2022). Its prevalence was estimated at approximately 1 in 8500 to 25,000 live births (de Mollerat et al. 2003). To date, at least six SHFM loci (SHFM1‐6) have been identified (Online Mendelian Inheritance in Man 2022; Umair and Hayat 2020), among which SHFM4 (OMIM# 605289) is caused by heterozygous mutations in the *TP63* (tumor protein p63) gene.

**FIGURE 1:**
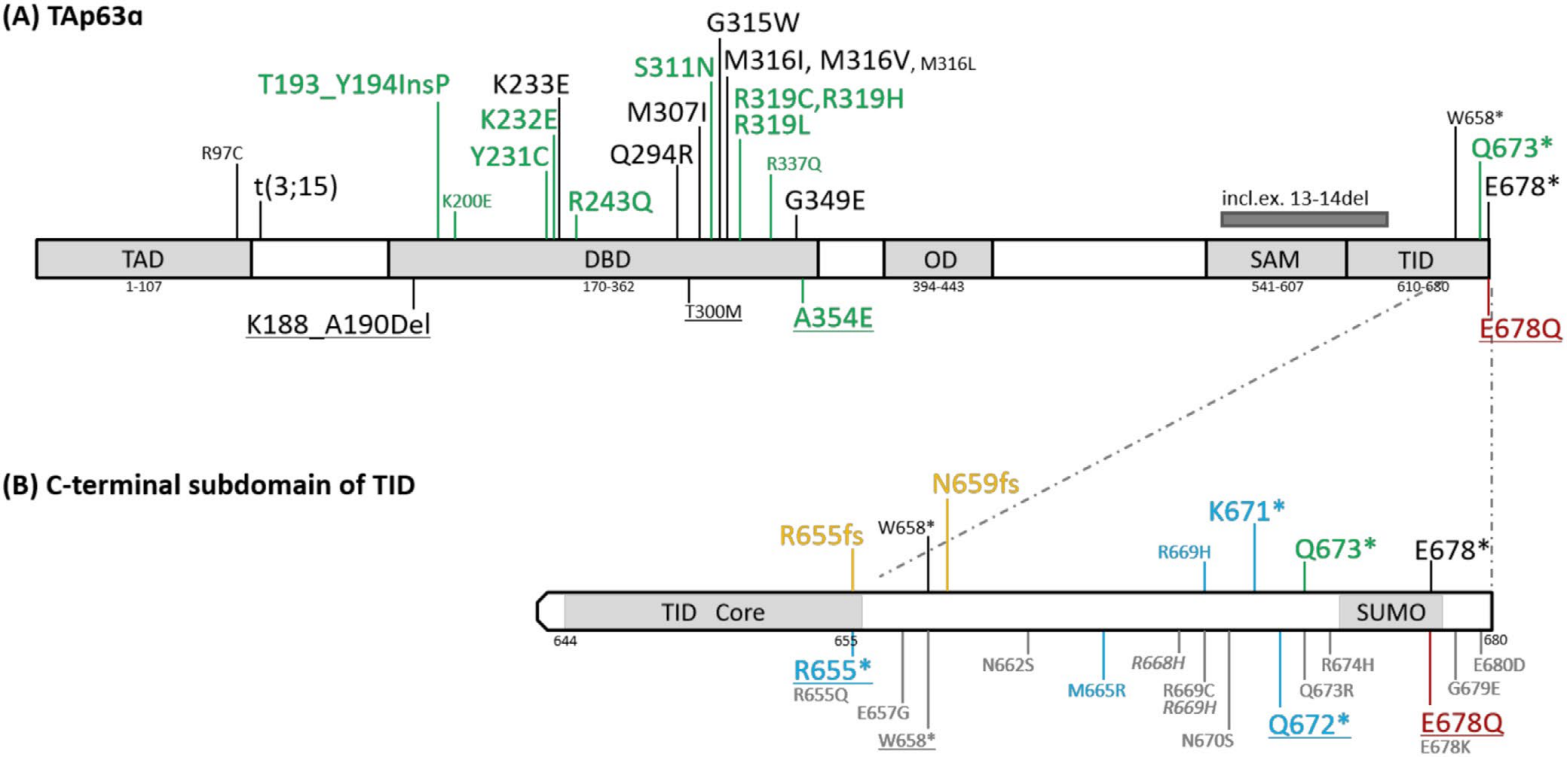
Spectrum of SHFM4 mutations in the TAp63α isoform (UniProtKB: Q9H3D4). (A) SHFM4 mutations cluster in two regions: the DBD and the C‐terminal subdomain of TID (residues 656–680). Both regions harbor SHFM4‐only mutations (black/red) and dual‐phenotype SHFM4/EEC3 mutations (green). The C‐terminal subdomain of TID includes two relatively reliable SHFM4‐only mutations at E678 within the sumoylation motif. (B) Close‐up of the C‐terminal subdomain of TID. Mutations are annotated by phenotype: SHFM4‐only (black), novel SHFM4‐only (red, this study), dual SHFM4/EEC3 (green), EEC3 only (blue), AEC/RHS (yellow), or undefined (gray). Smaller font indicates lower phenotypic reliability. Variants are sourced from HGMD (top) and ClinVar (bottom; underlined: (likely) pathogenic; italic: likely benign). AEC, ankyloblepharon‐ectodermal defects‐cleft lip/palate syndrome; DBD, DNA binding domain; EEC3, ectrodactyly‐ectodermal dysplasia‐cleft lip/palate syndrome 3; OD, oligomerization domain; RHS, Rapp–Hodgkin syndrome; SAM, sterile alpha motif domain; SHFM4, split‐hand/foot malformation 4; SUMO, sumoylation motif; TAD, transactivation domain; TID, transactivation inhibitory domain.

The *TP63* gene encodes the p63 protein, a member of the p53 transcription factor family. p63 plays a critical role in the development of stratified epithelium and the maintenance of genetic integrity of oocytes (Osterburg et al. 2021). The protein exists in multiple isoforms, with the longest isoform, TAp63α (Q9H3D4, 680aa), containing at least the following functional domains: a transactivation domain (TAD), a DNA binding domain (DBD), an oligomerization domain, a sterile alpha motif (SAM) domain, and a transactivation inhibitory domain (TID) (Figure 1) (UniProt Consortium 2023). The TID, located at the C‐terminus, includes a sumoylation motif in its C‐terminal subdomain, which is essential for post‐translational modification (PTM) and protein stability (Straub et al. 2010).

Heterozygous mutations in *TP63* are associated with a spectrum of syndromic and non‐syndromic disorders. These conditions are characterized by various combinations of ectodermal dysplasia, SHFM/ectrodactyly, cleft lip/palate, lacrimal duct obstruction, hypo‐ or hyper‐pigmentation, hypoplastic breasts, hypospadias, etc. (Sutton and van Bokhoven 2010). The ectrodactyly‐ectodermal dysplasia‐cleft lip/palate syndrome 3 (EEC3, OMIM# 604292) is considered the prototype of *TP63*‐related disorders and is defined by the triad of ectrodactyly, ectodermal dysplasia, and cleft lip/palate. At the same time, EEC3 exhibits significant inter‐ and intra‐familial phenotypic variability, and its phenotypic spectrum can encompass nearly all symptoms associated with *TP63* mutations. EEC3 cases presenting the classic triad are relatively rare, and SHFM/ectrodactyly is the most frequently observed feature (Rinne et al. 2006).

Most EEC3‐causing mutations are missense mutations within the DBD (Sutton and van Bokhoven 2010; Rinne et al. 2006). However, similar or identical mutations in the DBD also lead to limb‐mammary syndrome (LMS, MIM#603543) and acro‐dermato‐ungual‐lacrimal‐tooth syndrome (ADULT syndrome, MIM#103285). Due to overlapping phenotypes and genotypes, these three syndromes are often difficult to distinguish and have been proposed to share a unified nomenclature (Prontera et al. 2011; Osterburg et al. 2021). Here we collectively refer to them as “EEC3‐like syndrome” or simply “EEC3” unless otherwise specified.

According to the Human Gene Mutation Database (HGMD Professional 2024.3), 21 *TP63* mutations have been associated with the “Split hand/foot malformation” phenotype (Stenson et al. 2020). These mutations are primarily clustered in two regions: the DBD, which harbors more than 10 missense mutations, and the TID, which contains three nonsense mutations (Figure 1A). Some SHFM4 mutations in DBD have exhibited significant phenotypic variation, not only in the severity of limb malformation but also in the development of other abnormalities within the EEC3 spectrum. For example, the SHFM4 mutation K232E has been reported to cause isolated SHFM, isolated ectodermal dysplasia, ectrodactyly‐ectodermal dysplasia, and typical EEC3 within a single family (Wei et al. 2012). Such mutations that cause both isolated SHFM4 and EEC3‐like syndromes can be called “SHFM4/EEC3 mutations”. However, the full number and distribution of SHFM4/EEC3 mutations remain unknown, particularly, the phenotypic variability of SHFM4 mutations in the TID have not been systematically discussed.

We also noted that certain *TP63* mutations in HGMD have atypical phenotypic descriptions potentially representing isolated SHFM4, and some submission/condition records for *TP63* variants in ClinVar were based on the same case as the HGMD records. These observations further complicate the SHFM4 spectrum delineation.

In this study, we identified a novel *TP63* missense mutation within the sumoylation motif near the protein C‐terminus in a Chinese family with isolated SHFM. This represents the first missense SHFM4‐causing mutation in the TID region. We systematically compiled all reported *TP63* mutations causing isolated SHFM4, redefined their phenotypic classifications, and discussed their phenotypic patterns and structural features, with particular focus on the sumoylation‐related, C‐terminal subdomain of the TID.

## 2 Case Presentation

### 2.1 Phenotypes of the Pedigree

**FIGURE 2:**
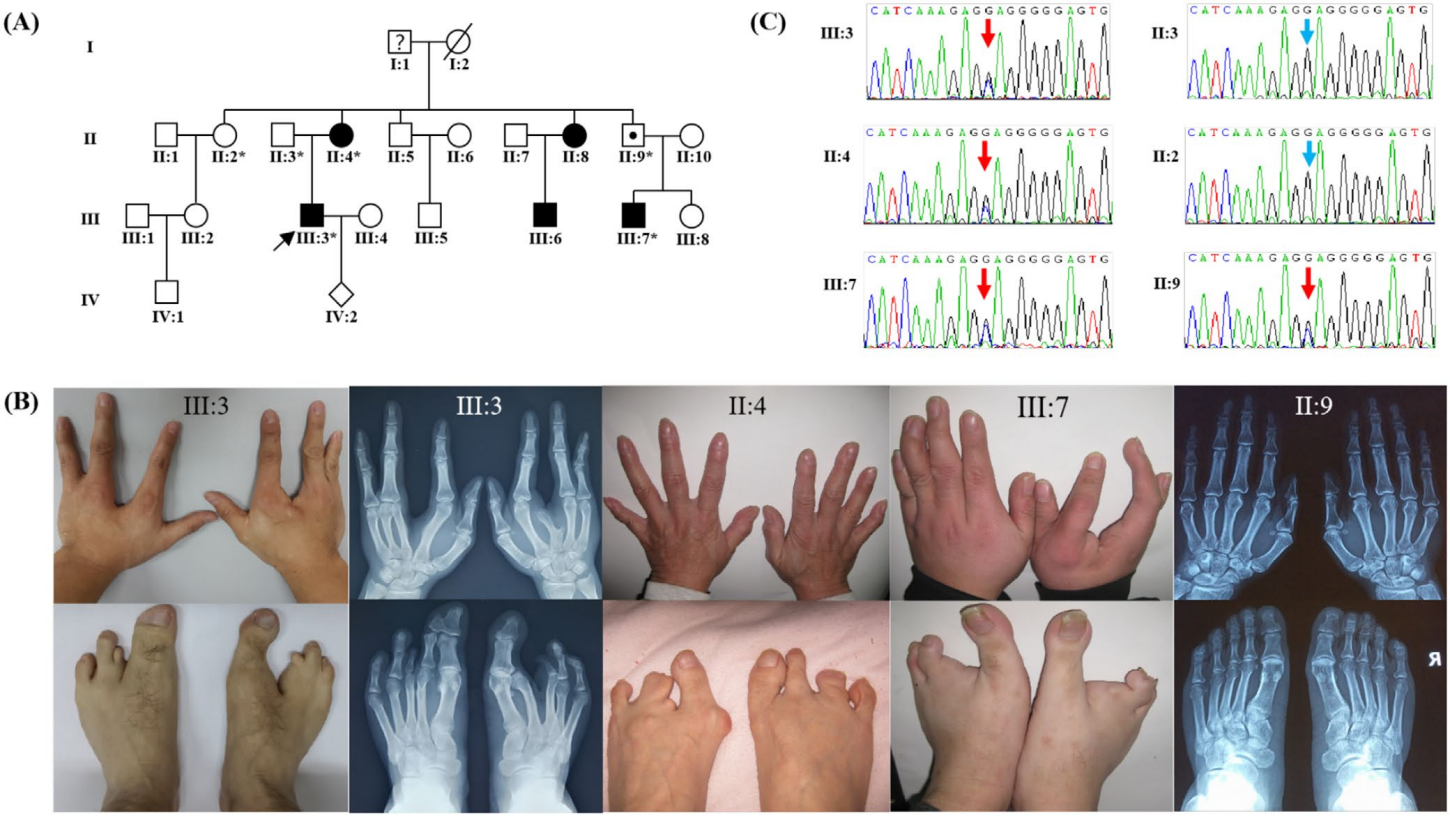
A Chinese family with *TP63*‐associated split hand/foot malformation. (A) Four‐generation pedigree. Arrow: the proband; Asterisks: sampled individuals. (B) Phenotypic spectrum of split hand/ft malformations associated with novel *TP63* mutation. Radiographs of obligate carrier (II:9) demonstrate non‐penetrance. (C) Sanger chromatograms reveal heterozygous c.2032G>C (p.E678Q) mutation in affected members (III:3, II:4, III:7) and obligate carrier (II:9), while not in unaffected family member (II:2, II:3).

A 28‐year‐old male was referred to our laboratory for genetic evaluation of familial limb deformities. He stated that the deformity did not significantly impact his daily life and that he was employed in an office‐based position. Physical examination revealed central ectrodactylies in all extremities, clinodactylies/camptodactylies of the bilateral 5th fingers, and syndactyly of the 1st and 2nd toes on the left foot (Figure 2B).

X‐ray imaging of the hands showed bilateral absence of the 3rd fingers, an abnormal joint formation between the 3rd metacarpal and the 4th proximal phalange of the left hand, and a short forklike deformity of the right 3rd metacarpal. Foot X‐rays revealed the absence of phalanges in the left 3rd toe, the entire right 2nd toe, and the distal phalange of the right 3rd toe, as well as osseous syndactyly between the distal phalanges of the 1st and 2nd toes on the left foot. No significant abnormalities were observed in bone structure, joint space, or soft tissue (Figure 2B).

The patient described that the limb deformities were transmitted among his maternal relatives and were traceable over four generations (Figure 2A). The inheritance pattern was consistent with autosomal dominance.

Phenotype data of other family members were collected primarily through photographs, X‐rays, and textual or verbal descriptions, as face‐to‐face examinations were not available. Marked phenotypic variation was observed (Figure 2B). The proband's mother (II:4) exhibited milder deformities, including bilateral camptodactylies of the 5th fingers, bilateral syndactylies of the 3rd and 4th toes, and a suspected absence of the left 2nd toe. One of the proband's cousins (III:7) displayed pronounced central clefts in three extremities and multiple camptodactylies/clinodactylies. In contrast, individual II:9, the father of III:7, had normal hands and feet confirmed by X‐rays, indicating a case of non‐penetrance. An aunt (II:8) and her son (III:6) were reported to have ectrodactylies in their feet, though no photographs were available. The proband's offspring (Individual IV:2) was born after the causative mutation had been identified and the prenatal targeted genetic testing had yielded a negative result.

No other significant congenital abnormalities, such as long bone deficiency, hearing loss, ectodermal dysplasia, cleft lip/plate, lacrimal duct obstruction, or hypoplastic breasts, were reported in the family. The menopausal age for individuals II:4 and II:8 was approximately 50 years.

Based on the clinical features observed in the family members, a tentative diagnosis of isolated SHFM was proposed.

### 2.2 Genetic Analysis

Peripheral blood samples were collected from six family members (Figure 2A), and genomic DNA was extracted. Based on the dominant inheritance pattern and molecular characteristics of SHFMs, we prioritized analysis of two loci: *TP63* point mutations (SHFM4) and 10q24 copy number variations (SHFM3, OMIM# 246560), both of which can be efficiently detected with traditional methods.

Sanger sequencing of the *TP63* gene (methods as in Shen et al. 2018) and real‐time quantitative polymerase chain reaction (qPCR) of 10q24 genomic fragments were performed on the proband. While no copy number variation was detected at the SHFM3 locus, Sanger sequencing revealed a heterozygous missense variant (NM_003722.5: c.2032G>C, p.Glu678Gln [E678Q]) in *TP63* that segregated perfectly with the phenotype: present in all affected individuals (III:3, II:4, III:7) and the asymptomatic obligate carrier (II:9), but absent in unaffected members (II:2, II:3) (Figure 2C).

This variant has not been recorded in healthy population databases (gnomAD and dbSNP) or disease databases (ClinVar and HGMD). The affected residue (E678) is conserved within the C‐terminal sumoylation consensus motif. Notably, a SHFM4‐causing nonsense mutation at the same position (c.2032G>T, p.E678*) is documented in HGMD. In silico analysis using VarCards (integrating SIFT, Polyphen‐2, LRT, M‐CAP, etc.) yielded consistently deleterious predictions (damaging score = 0.87; Li et al. 2018). According to the ACMG/AMP guidelines, the variant was classified as “likely pathogenic” (PM1: located in a mutational hotspot and/or critical and well‐established functional domain without benign variation; PM2: absent from controls; PP1: co‐segregation; PP3: multiple lines of computational evidence supporting a deleterious effect). Therefore, the diagnosis of isolated SHFM4 was confirmed, and the mutation has been deposited in ClinVar (Accession: VCV003233406.1).

## 3 Literature Review

To redefine the genotype and phenotype spectrum of SHFM4, we systematically reviewed all reported *TP63* mutations causing non‐syndromic/isolated SHFM, reassessing their phenotypic evidence. Our tripartite search included: (1) screening the HGMD database (Professional 2024.3) for all (probable) disease‐causing mutation potentially causing isolated SHFM4 phenotypes, across *TP63* isoforms (*TP63* [NM_003722.5], *tp63dn* [NM_001114980.2], and *tp63i3* [NM_001114979.2]); (2) quering ClinVar for *TP63* variants with condition records of “Split hand‐foot malformation 4” or “ectrodactyly”; (3) performing PubMed searches using the terms “TP63” and “split hand foot” which did not yield additional mutations. Pathogenicity assessments followed the ACMG/AMP 2015 guideline (Richards et al. 2015). Priority was given to records in public databases for conflicting case. Mutations classified as (likely) pathogenic/(probable) disease‐causing were retained in the SHFM4 mutation list.

We identified 28 distinct *TP63* mutations including 24 from HGMD (Professional 2024.3) and 4 with SHFM4 designation exclusively in ClinVar (Figure 1A; Table S1). These mutations were reported in 72 families (182 carriers), among which 62 families (158 patients) had phenotypic records sufficiently clear for classification as isolated SHFM or syndromic/non‐isolated forms. Based on phenotypic manifestations, these SHFM4 mutations were classified into two principal categories (Figure 1A).

- SHFM4‐only mutations (*n* = 16):
  ○ *R*elatively r*eliable SHFM4‐only mutations* (*n* = 11) exhibited isolated SHFM without evidence of *TP63‐*related extra‐limb anomalies.
  ○ *Less reliable SHFM4‐only mutations* (*n* = 5) showed heterogeneous uncertainties (Table S1): W658* carrier(s) had overgeneralized phenotype record(s) suggesting plausible but unconfirmed extra‐limb risks; the sole c.(?1653–65)(1873_?)del carrier only had prenatal ultrasound assessment, precluding comprehensive phenotype evaluation due to age limitations; R97C or T300M carriers displayed extra‐limb abnormalities lacking definitive *TP63* attribution; and M316L manifested only nonspecific “abnormality of the skeletal system” although the same‐residue variants (M316I/V) consistently caused isolated SHFM4.
- SHFM4/EEC3 mutations (*n* = 12):
  ○ *Reliable SHFM4/EEC3 mutations* (*n* = 10) demonstrated clear dual SHFM4/EEC3 phenotypes.
  ○ *Unreliable SHFM4/EEC3 mutations* (*n* = 2) predominantly caused EEC3‐like syndrome, with questionable SHFM4 association: K200E's sole case exhibited a syndromic phenotype within the EEC3 spectrum, yet it is retained in the SHFM4 list due to HGMD classification; R337Q's single putative SHFM4 case (also by prenatal ultrasound diagnosis) could not exclude developing ectodermal dysplasia.

The phenotypic and distribution characteristics of these SHFM4 mutations are analyzed below.

## 4 Discussion

### 4.1 Refinements to SHFM4 Mutation Identification Criteria

By incorporating multiple *TP63* isoforms, atypical phenotype descriptions, and thorough reviews of original reports, we established an expanded spectrum of SHFM4 mutations.

The identified HGMD phenotype terms for these mutations include: (1) “Split hand/foot malformation” (21 mutations); (2) “Split‐hand/split‐foot malformation” (2 mutations, 1 overlapping with category 1); (3) “Hand oligodactyly and toe syndactyly” (initially attributed to M316V in *tp63i3* but verified in the canonical *TP63* isoform); (4) “Abnormality of the skeletal system” (M316L). Variants with only non‐specific phenotype descriptions (e.g., “Fetal abnormalities”, “*TP63*‐related disorder”) were excluded.

Two *tp63i3* mutations (NM_001114979:c.1412C>A [p.S471Y] and c.1417G>A [p.V473I]) were documented in HGMD as probable disease‐causing mutations, but were excluded due to: (1) atypical “polydactyly” phenotypes, and (2) lacking functional validation and supportive computational evidence (VarCards damaging score = 0.62 for S471Y, 0.52 for V473I) (Zu et al. 2021). A *TP63* variant NM_003722: c.1825G>A (p.E609K) was excluded due to insufficient pathogenicity evidence: consistently classified as variants of uncertain significance (VUS) in ClinVar (Accession: VCV000069817.5) and subthreshold computational prediction scores (VarCards = 0.65; AlphaMissense = 0.515) (Li et al. 2018; Cheng et al. 2023).

### 4.2 Phenotypic Characteristics of SHFM4 Mutations

Our analysis revealed a substantial proportion of SHFM4/EEC3 mutations, which highlights the considerable phenotypic variability associated with SHFM4 mutations. Despite this intrinsic overlap, we identified specific phenotypic features in SHFM4 mutation carriers that diverge from classic EEC3 presentations.

According to our analysis, SHFM4 mutations (*n* = 152) and EEC3‐like syndrome mutations share the same triad symptom hierarchy (ectrodactyly>ectodermal dysplasia>cleft lip/palate), though with differential frequencies (SHFM4: 91%, 30%, 7%; EEC3: 68%, 50%, 37%) and dissimilar ectodermal dysplasia patterns (Table S1; Rinne et al. 2006). In EEC3, hair, lacrimal gland, nail, and tooth abnormalities occur at comparable frequencies of (51%–55%), whereas skin and breast manifestations are less common (both 34%) (Rinne et al. 2006). By contrast, patients with SHFM4 mutation (*n* = 139) exhibit tooth abnormalities as the predominant feature (25%), followed by skin (19%), hair (19%), and nail (18%) involvement, with lacrimal gland (6%) and breast (4%) abnormalities being rare. Notably, exclusion of the recurrent “unreliable” SHFM4/EEC3 mutation R337Q yielded a refined SHFM4 cohort (*n* = 137 for triad symptoms; *n* = 125 for ectodermal sub‐phenotypes) showing modified profiles: the overall ectodermal dysplasia frequency decreased to 23% (32/137), where tooth (18%), hair (14%), skin (11%) and nail (10%) abnormalities predominated, and lacrimal gland (3%)/breast (0%) involvement became negligible.

Importantly, the non‐penetrance rate in EEC3 patients remains undocumented, whereas we observed approximately 7.7% (14/182) non‐penetrance in SHFM4 mutation carriers, with a higher incidence in SHFM4‐only (9.4%, 6/64) versus SHFM4/EEC3 carriers (6.8%, 8/118) (Table S1).

In the present E678Q family, individual II:9 represents a non‐penetrant case. Furthermore, unverified family history suggested ectrodactyly in several nephews of patient I:1, but further details were unavailable for confirmation. Therefore, this branch was omitted from the pedigree diagram, and individual I:1 may either be non‐penetrant or have subtle malformations (Figure 2A).

### 4.3 Structural Basis for SHFM4 (−Only) Mutations

The genotype–phenotype relationship in SHFM4 remains poorly defined compared to other *TP63*‐related disorders (Sutton and van Bokhoven 2010; Osterburg et al. 2021). The reported distribution of SHFM4 mutations—either scattered throughout p63 or clustered in TAD and DBD—may reflect inclusion of mutations with less reliable phenotypic identification (Osterburg et al. 2021; Sutton and van Bokhoven 2010).

The R97C variant represents the sole SHFM4(‐only) mutation in TAD, but its classification is “less reliable” due to: (1) one HGMD entry documents an extra‐limb abnormality while another completely lacks phenotypic data (Table S1); (2) ClinVar latest submissions classify it as VUS for “*TP63*‐Related Spectrum Disorders” or multiple conditions (Table S1); (3) other R97 variants (R97H/P) consistently exhibit non‐SHFM4 phenotypes (ClinVar Accession: VCV000596662.11, VCV001677243.2).

Following our classification, the 11 relatively reliable SHFM4‐only mutations (excluding the translocation) are exclusively localized to either the DBD or the C‐terminal subdomain of TID. For DBD‐localized SHFM4‐only mutations, we observe a distinctive pattern: although occupying similar secondary structural motifs as EEC3 and/or SHFM4/EEC3 mutations, the SHFM4‐only mutations uniformly spare all DNA‐contacting residues (S311, R318, R343, A346, C347, R350) and Zinc‐coordinating sites (H247, C308, C312). In contrast, these canonical functional sites are frequently affected in EEC3 mutations (Osterburg et al. 2023; Stenson et al. 2020) and occasionally affected in SHFM4/EEC3 mutations. The graded disruption frequencies (EEC3 > SHFM4/EEC3 > SHFM4‐only) exhibit clear correspondence with their phenotypic continuum.

Despite the predominant ‘negative signature’ of the SHFM4‐only mutations, some notable features emerge: (1) The K233E mutation, reported in the largest SHFM4 family, may alter a potential PTM site (Pokorná et al. 2021; Jeon et al. 2012); (2) Recurrent SHFM4‐only mutations at M316 (M316I/V/L) and the adjacent G315W suggest a possible SHFM4‐only mutation hotspot with potential structural specificity; (3) The t(3;15) (q28;q21) translocation likely disrupts limb development through a breakpoint between ΔNp63's promoter and enhancer region, with functional studies confirming ΔNp63ɑ's essential role in SHFM pathogenesis (Table S1; Pokorná et al. 2021; Lo Iacono et al. 2008; Restelli et al. 2014). These findings collectively indicate that while SHFM4‐only mutations share a common avoidance of classical DBD functional sites, certain mutations may possess additional variant‐specific molecular attributes that could drive their pathogenesis.

### 4.4 Genotypes and Phenotypes in C‐Terminal Subdomain of TID

The C‐terminal subdomain of TID, containing the last 25 amino acids of the TAp63ɑ isoform, is the other hotspot for SHFM4 mutations. Structural predictions consistently identify this subdomain as an intrinsically disordered region lacking stable structure, evidenced by uniformly low confidence scores in modeling (e.g., model AF‐Q9H3D4‐F1‐v4 by AlphaFold, https://alphafold.com/entry/Q9H3D4) (Jumper et al. 2021; Varadi et al. 2022). Within the subdomain, residues 675–678 (IKEE) form the canonical sumoylation consensus motif, ψKΧD/E, where ψ is a large hydrophobic residue and X is any amino acid residue (Huang et al. 2004; Celen and Sahin 2020). SUMO (small ubiquitin‐like modifier) protein can be conjugated to K676, facilitating p63 degradation in most studies (Straub et al. 2010; Ghioni et al. 2005). However, many aspects of this process remain poorly understood, such as isoform specificity, SUMO paralog involvement, and crosstalk with other PTMs (Celen and Sahin 2020; Kawata et al. 2017; Vivo et al. 2009; Ranieri et al. 2018). These knowledge gaps significantly hinder the elucidation of the pathogenic mechanisms underlying related mutations (Restelli et al. 2014).

Most (likely) pathogenic mutations in the subdomain are nonsense mutations that remove the entire IKEE motif, except for Q678* (Figure 1B). These nonsense mutations have caused either SHFM4/EEC3 dual phenotypes (Q673*; Table S1), a suspected SHFM4‐only phenotype (W658*; Table S1), or pure EEC3 phenotypes (R655*, K671*, Q672*; ClinVar Accession: VCV002434205.3; Rinne et al. 2006; ClinVar Accession: VCV002710369.2), forming an EEC3‐like spectrum with high variability.

In contrast, the two “relatively reliable” SHFM4‐only mutations, E678Q and E678*, neither remove the entire IKEE motif nor disrupt the critical SUMO acceptor residue K676. Instead, they specifically affect the conserved E678, the 4th residue within the motif. In vitro studies showed that the E678* mutant lost affinity to the SUMO E2 conjugating enzyme, while a K676E artificial mutant did not (Huang et al. 2004). Additionally, the E678* mutant was partially resistant to SUMO1‐induced protein instability, unlike the almost fully resistant artificial K676R (Ghioni et al. 2005). These findings indicate that disrupting E678 affects sumoylation differently from disrupting K676, potentially providing a specific structural basis. More specifically, substituting the hydrophilic, negatively charged glutamic acid (E678) with the hydrophilic but uncharged glutamine (Q) may have a unique effect on the PTM, leading to the isolated SHFM phenotype.

Thus, as the first missense SHFM4‐causing mutation in the TID, with a potential unique structural basis and multiple patients of isolated SHFM4, E678Q stands out as the most reliable SHFM4‐only mutation in this region.

To date, only two (possibly) pathogenic missense variant in this subdomain, E678Q and R669H, have been documented in disease databases. The R669H has two inconsistent records in HGMD: one reports an “EEC3” patient and the other reports a “hypotonia” patient (Stranneheim et al. 2021; Ek et al. 2023). Moreover, it's latest evaluation in ClinVar is “likely benign” with a “not provided” condition (Accession: VCV000722833.4). Over 10 missense VUS in this subdomain are documented in ClinVar (Figure 1B). According to the AlphaMissense model (AF‐Q9H3D4‐F1‐v4), most of them demonstrate low pathogenicity potential, with a minor subset near the C‐terminus of the IKEE motif showing elevated pathogenic predictions (Cheng et al. 2023). Phenotypic descriptions of these VUS are overly general or lacking, such as “TP63‐related spectrum disorders”, “Inborn genetic diseases”, “not provided”. In summary, except for the E678Q, the pathogenicity and phenotypes of these missense variants in this subdomain remain unclear.

Additionally, small frameshift deletions at the N‐terminus of the sumoylation‐related subdomain caused ankyloblepharon‐ectodermal defects‐cleft lip/palate syndrome (AEC, OMIM #106260) or Rapp–Hodgkin syndrome (RHS, OMIM #129400) (Figure 1B). SHFM rarely appears in their phenotypes. So far, frameshift mutations causing AEC/RHS are mainly restricted to the SAM domain and the N‐terminal portion of TID (Stenson et al. 2020), differing from the C‐terminal subdomain of TID discussed in this study. Their resulting mutant peptides have abnormally extended C‐termini that cause protein misfolding and aggregation, independent of the sumoylation (Russo et al. 2018). Given their distinct genotypes and phenotypes, SHFM4/EEC3‐like syndrome mutations and AEC/RHS mutations in this region are easily distinguishable.

## 5 Conclusion

We reported a novel missense mutation (E678Q) within the C‐terminal sumoylation motif of p63 that causes isolated SHFM4. Through comprehensive analysis of disease databases and literature review, we expanded the SHFM4 mutation spectrum to 28 *TP63* mutations, classified into 16 SHFM4‐only and 12 SHFM4/EEC3 variants, with further stratification based on reliability. Our findings highlight the sumoylation‐related C‐terminal subdomain as a hotspot for SHFM4 mutations, with E678Q being the most reliable SHFM4‐only mutation in this region to date.

This study provides valuable insights for improving genetic diagnosis and counseling for SHFM4‐related disorders.

## Author Contributions

W.Y. and X.Z. designed the study. W.Y. gathered clinical data from the patients. J.Z. performed the PCR and qPCR experiment. W.Y. and N.S. drafted the manuscript. X.Z. reviewed and revised the manuscript. All authors read and approved the final manuscript.

## Ethics Statement

This study was conducted in accordance with ethic approvals granted by the Institutional Review Board of Institute of Basic Medical Sciences, Chinese Academy of Medical Sciences (Project 005‐2009 approved on 2009‐03‐30; Project 2022170 approved on 2022‐06‐02). Written informed consent covering study participation and the use of de‐identified data/images for publication was obtained from all participants.

## Conflicts of Interest

The authors declare no conflicts of interest.

## Supporting information


**Table S1:** Phenotypic statistics of SHFM4 mutations.

## Data Availability

A summary of phenotypes of all SHFM4 mutations is available in the Supporting Information. All result data supporting the conclusions of this article are included within the article and its additional file. Genetic testing method can be provided by inquiring with the corresponding author.
