# Supplementary material for: A Novel TP63 Missense Mutation in the Sumoylation Motif Causes Isolated Split‐Hand/Foot Malformation 4: A Pedigree Report and Literature Review
Source: Mol Genet Genomic Med. 2025 Sep 18;13(9):e70140. doi: 10.1002/mgg3.70140 (PMC12444410; doi:10.1002/mgg3.70140)
Supplement: Supplementary file 1 — Table S1: Phenotypic statistics of SHFM4 mutations. [file MGG3-13-e70140-s001.docx]

**Supplementary Table 1. Phenotypic Statistics of SHFM4 Mutations:**

| Mutation & serial number | | Srutucture^a^ | Family | Phenotypic diagnosis^b^ | Patients^c^ (+ non-penetrant carriers) | Limb malformation^d^ | Cleft Lip/Palate | Ectodermal Dysplasia^e^ | | | | | | | Other Feature | Reference^f^ | |
| --- | --- | --- | --- | --- | --- | --- | --- | --- | --- | --- | --- | --- | --- | --- | --- | --- | --- |
|  |  |  |  |  |  |  |  | ED (total) | breast | Hair | lacrimal duct | Nail | skin | Teeth |  |  |  |
| Mutations in transactivation domain (TAD): | | | | | | | | | | | | | | | | |  |
| 1 | p.R97C | β-strand 87- 98 | 1-1 | SHFM? | 1 | 1 | - | ND | - | - | - | - | (ACC) | - | - | Zenteno et al. 2005 | |
|  |  |  | 1-2 | NI | 1 | NI | NI | NI |  |  |  |  |  |  | NI | Capalbo et al. 2019 | |
|  |  |  | 1-3 | TP63 | 1 | NI | NI | NI |  |  |  |  |  |  | NI | RCV005031401.1 | |
|  |  |  | 1-4 | TP63 | 1 | NI | NI | NI |  |  |  |  |  |  | NI | RCV001851711.7 | |
| Mutations in DNA binding domain (DBD): | | | | | | | | | | | | | | | | |  |
| 2 | c.563_571del (p.K188_A190del) | Loop 181- 191 | 2-1 | SHFM4 | 3 | 3 | - | - |  |  |  |  |  |  | - | RCV002244562.2 | |
| 3 | c.580-2A>C; p.T193_Y194insPro | β-strand 192- 195 | 3-1 | SHFM | 1 | 1 | - | - |  |  |  |  |  |  | - | van Bokhoven et al. 2001 | |
|  |  |  | 3-2 | EEC/EE | 2 | 2 | - | 2 | - | 2 | - | 2 | 2(Sd) | 1 | - | Kantaputra, Matangkasombut, and Sripathomsawat 2012 | |
| 4 | p.K200E | β-strand 200- 203 | 4-1 | EEC3(SHFM) | 1 | 1 | 1 | -/1(ND) |  |  |  |  |  |  | - | Simonazzi et al. 2012 | |
| 5 | p.Y231C | β-strand 224- 231 | 5-1^g^ | EEC/EE | 2 | 2 | - | 2 | NI | NI | NI | NI | NI | NI | - | van Bokhoven and Brunner 2002 | |
|  |  |  | 5-2^h^ | SHFM? | 2 | 2 | - | -/2(ND) | - | - | - | - | - | -/2(ND) | 2(high-arched palate) | He et al. 2017 | |
|  |  |  | 5-3 | SHFM | 1 | 1 | - | - |  |  |  |  |  |  | - | Jourdain et al. 2020 | |
| 6 | p.K232E | loop 232-233 /loop 232- 264 | 6-1 | SHFM | 2 | 2 | - | - |  |  |  |  |  |  | - | van Bokhoven et al. 2001 | |
|  |  |  | 6-2 | EEC | 10 | 9 | 1 | 6 | - | 6 | - | 1 | - | 5 | 2(hearing impairment), 3(facial abnormalities) | Wei et al. 2012 | |
| 7 | p.K233E | loop 232-233 /loop 232- 264 | 7-1 | SHFM | 21(+3) | 21 | - | - |  |  |  |  |  |  | - | Ianakiev et al. 2000 | |
| 8 | p.R243Q | loop 237-244 /loop 232- 264 | 8-1 | EEC | 1 | NI | NI | NI |  |  |  |  |  |  | NI | Celli et al. 1999 | |
|  |  |  | 8-2 | EEC | 1 | 1 | 1 | 1 | - | 1 | - | 1 | - | 1 | - | Yin et al. 2010 | |
|  |  |  | 8-3 | EEC/EE | 1 | 1 | - | 1 | - | - | - | - | 1(Sd) | 1 | 1(exfoliative dermatitis) | Otsuki et al. 2016 | |
|  |  |  | 8-4 | SHFM | 3 | 3 | - | - |  |  |  |  |  |  | - | Yang et al. 2018 | |
|  |  |  | 8-5 | EEC/EE | 2 | 2 | - | 2 | - | - | - | 2 | - | 2 | 2(cubitus valgus) | Zheng et al. 2019 | |
|  |  |  | 8-6 | EEC/EE | 1 | 1 | - | 1 | - | - | - | 1 | - | - | 1(renal pyelectasis; facial abnormalities; thorax skin outgrowth) | Hurni et al. 2021 | |
|  |  |  | 8-7 | SHFM4 | 1 | (1/syndactyly only?) | - | - |  |  |  |  |  |  | - | RCV002250454.1 | |
|  |  |  | 8-8 | TP63 | 1 | NI | NI | NI |  |  |  |  |  |  | NI | RCV000705452.7 | |
| 9 | p.Q294R | loop 290-299 | 9-1 | SHFM | 1 | 1 | - | - |  |  |  |  |  |  | - | Marinakis et al. 2021 | |

**Supplementary Table 1 (continued):**

| 10 | p.T300M | β-strand 300- 306 | 10-1 | SHFM4 | 1 | 1 | - | - |  |  |  |  |  |  | - | RCV003228727.1 |
| --- | --- | --- | --- | --- | --- | --- | --- | --- | --- | --- | --- | --- | --- | --- | --- | --- |
|  |  |  | 10-2 | Leukemia | 1 | NI | - | NI |  |  |  |  |  |  | Leukemia | Kim et al. 2021 |
| 11 | p.M307I | loop 307-320 | 11-1 | SHFM | 3(+1) | 3 | - | - |  |  |  |  |  |  | - | Miao et al. 2022 |
| 12 | p.S311N | loop 307-320 | 12-1 | EEC | 1 | NI | NI | NI |  |  |  |  |  |  | NI | Celli et al. 1999 |
|  |  |  | 12-2 | EEC | 1 | NI | NI | （1） | NI | NI | (1) | NI | NI | NI | limbal stem cell deficiency | Di et al. 2012 |
|  |  |  | 12-3 | SHFM | 1 | 1 | - | - |  |  |  |  |  |  | - | Moosa et al. 2022 |
| 13 | p.G315W | loop 307-320 | 13-1 | SHFM | 2(+1) | 2 | - | - |  |  |  |  |  |  | - | Yamoto et al. 2019 |
| 14 | p.M316I | loop 307-320 | 14-1 | SHFM | 2 | 2 | - | - |  |  |  |  |  |  | - | Geng et al. 2020 |
| 15 | p.M316L | loop 307-320 | 15-1 | Abnormality of skeletal system | 1 | NI | NI | NI |  |  |  |  |  |  | NI | Retterer et al. 2016 |
| 16 | p.M316V | loop 307-320 | 16-1 | SHFM | 2 | 2 | - | -/2(ND) |  |  |  |  |  |  | - | Fu et al. 2022 |
| 17 | p.R319C | loop 307-320 | 17-1 | SHFM | 17(+1) | 17 | - | - |  |  |  |  |  |  | - | Ianakiev et al. 2000 |
|  |  |  | 17-2 | EEC | 3 | 3 | 1(bifid uvula) | 3 | - | 3 | 3 | 3 | 3(Sd) | 3 | 1(hypospadias); 3(asthma) | Barrow et al. 2002 |
|  |  |  | 17-3 | EEC | 10 | 10 | 3 | 8 | - | 3 | - | - | 3(Sd) | 4 | - | Ray et al. 2004 |
|  |  |  | 17-4 | EEC/EE | 1 | 1 | - | 1 | - | - | - | - | - | 1 | - | Han et al. 2010 |
|  |  |  | 17-5 | EEC/EE | 1 | 1 | - | 1 | - | - | - | - | 1(Sp) | 1 | 1(congenital cataracts and glaucoma) | Pratsou et al. 2014 |
|  |  |  | 17-6 | EEC | 3(+1) | 3 | 2 | -/3(ND) |  |  |  |  |  |  | - | Yang et al. 2017 |
|  |  |  | 17-7 | EEC/EE | 1 | 1 | - | 1 | - | 1 | - | 1 | 1(Sp) | 1 | - | Zheng et al. 2019 |
|  |  |  | 17-8 | EEC3 | 1 | NI | NI | NI |  |  |  |  |  |  | NI | RCV003162215.2 |
|  |  |  | 17-9 | TP63 | 1 | NI | NI | NI |  |  |  |  |  |  | NI | RCV002512857.4 |
| 18 | p.R319H | loop 307-320 | 18-1 | SHFM | 1(+1) | 1 | - | - |  |  |  |  |  |  | - | van Bokhoven et al. 2001 |
|  |  |  | 18-2 | EEC | 1 | 1 | - | 1 | - | 1 | 1 | - | 1 | 1 | 1(eczema) | van Bokhoven et al. 2001 |
|  |  |  | 18-3 | SHFM | 1 | 1 | - | - |  |  |  |  |  |  | - | Carter et al. 2017 |
|  |  |  | 18-4 | SHFM | 3 | 3 | - | - |  |  |  |  |  |  | - | Bilal et al. 2020 |
|  |  |  | 18-5 | EEC/EE | 1(+1) | 1 | - | 1 | - | - | - |  | 1(Sp) | 1 | - | Otsuki et al. 2020 |
|  |  |  | 18-6 | SHFM？ | 2(+2) | 2 | - | -/2(ND) |  |  |  |  |  |  | - | Zhang et al. 2021 |
|  |  |  | 18-7 | TP63 | 1 | NI | NI | NI |  |  |  |  |  |  | NI | RCV002518757.4 |
|  |  |  | 18-8 | SHFM | 3(+2) | 3 | - | - |  |  |  |  |  |  | - | Zhuang et al. 2025 |
| 19 | p.R319L | loop 307-320 | 19-1 | SHFM | 1 | 1 | - | - |  |  |  |  |  |  | - | Carter et al. 2017 |
|  |  |  | 19-2 | EEC/ADULT | 1 | (1/syndactyly only) | - | 1 | - | 1 | - | 1 | 1(thin skin) | 1 | - | Yu et al. 2022 |
|  |  |  | 19-3 | EEC | 1 | 1 | 1 | - |  |  |  |  |  |  | - | Simpson et al. 2023 |
| 20 | p.R337Q | β-strand 334- 344 | 20-1 | ADULT | 8 | 3, (3/syndactyly only) | - | 8 | - | 4 | 4 | 8 | 7(Sp) | 6 | 8(neurodermitic signs) | Duijf et al. 2002 |
|  |  |  | 20-2^i^ | ADULT | 1 | 1 | - | 1 | 1 | - | - | - | 1(Sd & Sp) | 1 | - | Wang et al. 2009 |
|  |  |  | 20-3 | ADULT? ED? | 3 | - | - | 3 | 3 | 2 | 1 | 3 | 3(Sd & Sp) | 3 | - | Whittington et al. 2016 |

**Supplementary Table 1 (continued):**

|  |  |  | 20-4 | ADULT | 1 | (1/syndactyly only) | - | 1 | - | 1 | - | 1 | 1(thin skin) | 1 | - | Yu et al. 2022 | |
| --- | --- | --- | --- | --- | --- | --- | --- | --- | --- | --- | --- | --- | --- | --- | --- | --- | --- |
|  |  |  | 20-5 | SHFM？ | 1 | 1 | - | -/ND |  |  |  |  |  |  | - | Xu et al. 2023 | |
|  |  |  | 20-6 | ED | 1 | - | - | 1 | 1 | 1 | - | 1 | 1(Sd) | 1 | - | Zhou et al. 2023 | |
| 21 | p.G349E | Helix 348- 366 | 21-1 | SHFM | 5 | 5 | - | - |  |  |  |  |  |  | - | Luo et al. 2008 | |
| 22 | p.A354E | Helix 348- 366 | 22-1 | EEC | 1 | NI | NI | NI |  |  |  |  |  |  | NI | Rinne, Brunner, and van Bokhoven 2007 | |
|  |  |  | 22-2 | SHFM4 | 2 | 2 | - | - |  |  |  |  |  |  | - | RCV002290573.2 | |
|  |  |  | 22-3 | TP63 | 1 | NI | NI | NI |  |  |  |  |  |  | NI | RCV001049615.5 | |
| Mutations in Transactivation Inhibitory Domain (TID): | | | | | | | | | | | | | | | | |  |
| 23 | p.W658* | C-terminal of TID | 23-1 | SHFM | 1 | 1 | - | - |  |  |  |  |  |  | - | Sowińska-Seidler, Socha, and Jamsheer 2014 | |
|  |  |  | 23-2 | TP63 | 1 | NI | NI | NI |  |  |  |  |  |  | NI | RCV002046983.7 | |
| 24 | p.Q673* | C-terminal of TID | 24-1 | SHFM | 1 | 1 | - | - |  |  |  |  |  |  | - | van Bokhoven et al. 2001 | |
|  |  |  | 24-2 | EEC3 | 1 | NI | NI | NI |  |  |  |  |  |  | NI | RCV003330281.3 | |
| 25 | p.E678* | C-terminal of TID | 25-1 | SHFM | 1 | 1 | - | - |  |  |  |  |  |  | - | van Bokhoven and Brunner 2002 | |
| 26 | p.E678Q | C-terminal of TID | 26-1 | SHFM | 5(+1) | 5 | - | - |  |  |  |  |  |  | - | this report | |
| Gross Changes: | | | | | | | | | | | | | | | | |  |
| 27^j^ | c.(?_1653‐65)_(1873_?)del | SAM domain and TID | 27-1 | SHFM？ | 1 | 1 | - | -/ND |  |  |  |  |  |  | - | Jourdain et al. 2020 | |
| 28 | t(3;15) (q28;q21) | one breakpoint in intron 3 of TAp63α | 28-1 | SHFM | 3 | 3 | - | - |  |  |  |  |  |  | - | Peng et al. 2021 | |
| Total | |  | 72 |  | 168(+14) | 139(+6 syndactyly only) | 10 | 46 | 5 | 26 | 9 | 25 | 27 | 35 |  |  | |

^a^ Secondary structure of mutations in TAD and DBD predicted by AlphaFold (model AF-Q9H3D4-F1-v4). Numbers refer to residues in Q9H3D4.

^b^ Phenotype abbreviations: ADULT (acro-dermato-ungual-lacrimal-tooth); ED (ectodermal dysplasia); EE (ectrodactyly and ectodermal dysplasia); EEC (ectrodactyly-ectodermal dysplasia-cleft lip/palate syndrome); SHFM (Split hand/foot malformation); TP63 (TP63-related (Spectrum) disorders, or TP63-related multiple conditions). NI (no information). Parentheses show database discrepancies.

^c^ Unspecified patient numbers were counted as 1 per family.

^d^ Ectrodactyly or brachydactyly.

^e^ Ectodermal dysplasia (ED) manifestations: B (breast hypoplasia); H (sparse hair); L (lacrimal duct defects); N (nail dystrophy); Sd (dry skin); Sp (abnormal skin pigmentation); T (teeth hypodontia). ACC (aplasia cutis congenita) is defined as congenital localized absence of skin. "ND" (not determined) mostly result from limitations in prenatal ultrasound diagnosis.

^f^ Cases from ClinVar were given ID number of Variation/condition record.

^g^ Family 5-1 original report combined two Y231C families (both EE).

^h^ Family 5-2: high palatal arches and dental caries in both patients; father uniquely missing all mandibular teeth.

^i^ Family 20-2: additional members exhibited breast hypoplasia, hypohidrosis and dyspigmentation, but were mutation-negative.

^j^ Exons 13-14 deletion (boundaries unmapped) is expected to eliminate the C-terminal segment of sterile alpha motif domain and N-terminal portion of TID.

**References for Supplementary Table 1**

Barrow, L. L., van Bokhoven, H., Daack-Hirsch, S., Andersen, T., van Beersum, S. E., Gorlin, R., & Murray, J. C. (2002). Analysis of the p63 gene in classical EEC syndrome, related syndromes, and non-syndromic orofacial clefts. *Journal of medical genetics*, *39*(8), 559–566. https://doi.org/10.1136/jmg.39.8.559.

Bilal, M., Hayat, A., Umair, M., Ullah, A., Khawaja, S., Malik, E., Burmeister, M., Bibi, N., Umm-E-Kalsoom, Memon, M. I., Basit, S., Ahmad, W., & Khan, B. (2020). Sequence Variants in the *WNT10B* and *TP63* Genes Underlying Isolated Split-Hand/Split-Foot Malformation. *Genetic testing and molecular biomarkers*, *24*(9), 600–607. https://doi.org/10.1089/gtmb.2020.0024.

Capalbo, A., Valero, R. A., Jimenez-Almazan, J., Pardo, P. M., Fabiani, M., Jiménez, D., Simon, C., & Rodriguez, J. M. (2019). Optimizing clinical exome design and parallel gene-testing for recessive genetic conditions in preconception carrier screening: Translational research genomic data from 14,125 exomes. *PLoS genetics*, *15*(10), e1008409. https://doi.org/10.1371/journal.pgen.1008409.

Carter, T. C., Sicko, R. J., Kay, D. M., Browne, M. L., Romitti, P. A., Edmunds, Z. L., Liu, A., Fan, R., Druschel, C. M., Caggana, M., Brody, L. C., & Mills, J. L. (2017). Copy-number variants and candidate gene mutations in isolated split hand/foot malformation. *Journal of human genetics*, *62*(10), 877–884. https://doi.org/10.1038/jhg.2017.56.

Celli, J., Duijf, P., Hamel, B. C., Bamshad, M., Kramer, B., Smits, A. P., Newbury-Ecob, R., Hennekam, R. C., Van Buggenhout, G., van Haeringen, A., Woods, C. G., van Essen, A. J., de Waal, R., Vriend, G., Haber, D. A., Yang, A., McKeon, F., Brunner, H. G., & van Bokhoven, H. (1999). Heterozygous germline mutations in the p53 homolog p63 are the cause of EEC syndrome. *Cell*, *99*(2), 143–153. https://doi.org/10.1016/s0092-8674(00)81646-3.

Di Iorio, E., Kaye, S. B., Ponzin, D., Barbaro, V., Ferrari, S., Böhm, E., Nardiello, P., Castaldo, G., McGrath, J. A., & Willoughby, C. E. (2012). Limbal stem cell deficiency and ocular phenotype in ectrodactyly-ectodermal dysplasia-clefting syndrome caused by p63 mutations. Ophthalmology, 119(1), 74–83. https://doi.org/10.1016/j.ophtha.2011.06.044.

Duijf, P. H., Vanmolkot, K. R., Propping, P., Friedl, W., Krieger, E., McKeon, F., Dötsch, V., Brunner, H. G., & van Bokhoven, H. (2002). Gain-of-function mutation in ADULT syndrome reveals the presence of a second transactivation domain in p63. *Human molecular genetics*, *11*(7), 799–804. https://doi.org/10.1093/hmg/11.7.799.

Fu, F., Li, R., Yu, Q., Wang, D., Deng, Q., Li, L., Lei, T., Chen, G., Nie, Z., Yang, X., Han, J., Pan, M., Zhen, L., Zhang, Y., Jing, X., Li, F., Li, F., Zhang, L., Yi, C., Li, Y., … Liao, C. (2022). Application of exome sequencing for prenatal diagnosis of fetal structural anomalies: clinical experience and lessons learned from a cohort of 1618 fetuses. *Genome medicine*, *14*(1), 123. https://doi.org/10.1186/s13073-022-01130-x.

Geng, H., Tang, D., Xu, C., He, X., & Zhang, Z. (2020). A Novel Missense Variant of *TP63* Heterozygously Present in Split-Hand/Foot Malformation. *BioMed research international*, *2020*, 4215632. https://doi.org/10.1155/2020/4215632

Han, D., Wu, H., Zhang, X. X., & Feng, H. L. (2010). *Zhonghua kou qiang yi xue za zhi = Zhonghua kouqiang yixue zazhi = Chinese journal of stomatology*, *45*(12), 767–769.

He, W., Lin, G., Liang, P., Cheng, D., Hu, X., Zhou, L., Xiong, B., Tan, Y., Lu, G., & Li, W. (2017). *Zhonghua yi xue yi chuan xue za zhi = Zhonghua yixue yichuanxue zazhi = Chinese journal of medical genetics*, *34*(4), 476–480. https://doi.org/10.3760/cma.j.issn.1003-9406.2017.04.002.

Hurni, Y., Marangoni, M., Garofalo, G., Cassart, M., Tomasi, L., Vandernoot, I., Smits, G., & Gounongbé, C. (2021). Spontaneous resolution of nonimmune hydrops fetalis in a fetus with *TP63* gene mutation and *LZTR1* gene variants. *Clinical case reports*, *9*(8), e04624. https://doi.org/10.1002/ccr3.4624.

Ianakiev, P., Kilpatrick, M. W., Toudjarska, I., Basel, D., Beighton, P., & Tsipouras, P. (2000). Split-hand/split-foot malformation is caused by mutations in the p63 gene on 3q27. *American journal of human genetics*, *67*(1), 59–66. https://doi.org/10.1086/302972.

Jourdain, A. S., Petit, F., Odou, M. F., Balduyck, M., Brunelle, P., Dufour, W., Boussion, S., Brischoux-Boucher, E., Colson, C., Dieux, A., Gérard, M., Ghoumid, J., Giuliano, F., Goldenberg, A., Khau Van Kien, P., Lehalle, D., Morin, G., Moutton, S., Smol, T., Vanlerberghe, C., … Escande, F. (2020). Multiplex targeted high-throughput sequencing in a series of 352 patients with congenital limb malformations. *Human mutation*, *41*(1), 222–239. https://doi.org/10.1002/humu.23912.

Kantaputra, P. N., Matangkasombut, O., & Sripathomsawat, W. (2012). Split hand-split foot-ectodermal dysplasia and amelogenesis imperfecta with a TP63 mutation. *American journal of medical genetics. Part A*, *158A*(1), 188–192. https://doi.org/10.1002/ajmg.a.34356.

Kim, J., Gianferante, M., Karyadi, D. M., Hartley, S. W., Frone, M. N., Luo, W., Robison, L. L., Armstrong, G. T., Bhatia, S., Dean, M., Yeager, M., Zhu, B., Song, L., Sampson, J. N., Yasui, Y., Leisenring, W. M., Brodie, S. A., de Andrade, K. C., Fortes, F. P., Goldstein, A. M., … Mirabello, L. (2021). Frequency of Pathogenic Germline Variants in Cancer-Susceptibility Genes in the Childhood Cancer Survivor Study. *JNCI cancer spectrum*, *5*(2), pkab007. https://doi.org/10.1093/jncics/pkab007.

Luo, T., Yu, W., Yuan, Z., Deng, Y., Zhao, Y., Yuan, W., Xiao, J., Wang, Y., Luo, N., Mo, X., Li, Y., Liu, M., & Wu, X. (2008). A novel mutation of p63 in a Chinese family with inherited syndactyly and adactylism. *Mutation research*, *637*(1-2), 182–189. https://doi.org/10.1016/j.mrfmmm.2007.08.010.

Marinakis, N. M., Svingou, M., Veltra, D., Kekou, K., Sofocleous, C., Tilemis, F. N., Kosma, K., Tsoutsou, E., Fryssira, H., & Traeger-Synodinos, J. (2021). Phenotype-driven variant filtration strategy in exome sequencing toward a high diagnostic yield and identification of 85 novel variants in 400 patients with rare Mendelian disorders. *American journal of medical genetics. Part A*, *185*(8), 2561–2571. https://doi.org/10.1002/ajmg.a.62338.

Miao, M., Lu, S., Sun, X., Zhao, M., Wang, J., Su, X., Jin, B., & Sun, L. (2022). Identification of a novel heterozygous missense TP63 variant in a Chinese pedigree with split-hand/foot malformation. *BMC medical genomics*, *15*(1), 157. https://doi.org/10.1186/s12920-022-01311-y.

Moosa, S., Coetzer, K. C., Lee, E., & Seo, G. H. (2022). Undiagnosed disease program in South Africa: Results from first 100 exomes. *American journal of medical genetics. Part A*, *188*(9), 2684–2692. https://doi.org/10.1002/ajmg.a.62847.

Otsuki, Y., Ueda, K., Nuri, T., Satoh, C., Maekawa, R., & Yoshiura, K. I. (2020). EEC-LM-ADULT syndrome caused by R319H mutation in TP63 with ectrodactyly, syndactyly, and teeth anomaly: A case report. *Medicine*, *99*(44), e22816. https://doi.org/10.1097/MD.0000000000022816.

Otsuki, Y., Ueda, K., Satoh, C., Maekawa, R., Yoshiura, K. I., & Iseki, S. (2016). Intermediate Phenotype between ADULT Syndrome and EEC Syndrome Caused by R243Q Mutation in TP63. *Plastic and reconstructive surgery. Global open*, *4*(12), e1185. https://doi.org/10.1097/GOX.0000000000001185.

Peng, Y., Yang, S., Xi, H., Hu, J., Jia, Z., Pang, J., Liu, J., Yu, W., Tang, C., & Wang, H. (2021). Whole genome sequencing reveals translocation breakpoints disrupting TP63 gene underlying split hand/foot malformation in a Chinese family. *Molecular genetics & genomic medicine*, *9*(3), e1604. https://doi.org/10.1002/mgg3.1604.

Pratsou, P., Defty, C. L., Ozoemena, L., McGrath, J. A., Moss, C., & Gach, J. E. (2014). Limited ectrodactyly, ectodermal dysplasia and cleft lip-palate syndrome with a p63 mutation, associated with linear and whorled naevoid hypermelanosis. *Clinical and experimental dermatology*, *39*(2), 266–268. https://doi.org/10.1111/ced.12259.

Ray, A. K., Marazita, M. L., Pathak, R., Beever, C. L., Cooper, M. E., Goldstein, T., Shaw, D. F., & Field, L. L. (2004). TP63 mutation and clefting modifier genes in an EEC syndrome family. *Clinical genetics*, *66*(3), 217–222. https://doi.org/10.1111/j.1399-0004.2004.00287.x.

Retterer, K., Juusola, J., Cho, M. T., Vitazka, P., Millan, F., Gibellini, F., Vertino-Bell, A., Smaoui, N., Neidich, J., Monaghan, K. G., McKnight, D., Bai, R., Suchy, S., Friedman, B., Tahiliani, J., Pineda-Alvarez, D., Richard, G., Brandt, T., Haverfield, E., Chung, W. K., … Bale, S. (2016). Clinical application of whole-exome sequencing across clinical indications. *Genetics in medicine : official journal of the American College of Medical Genetics*, *18*(7), 696–704. https://doi.org/10.1038/gim.2015.148.

Rinne, T., Brunner, H. G., & van Bokhoven, H. (2007). p63-associated disorders. *Cell cycle (Georgetown, Tex.)*, *6*(3), 262–268. https://doi.org/10.4161/cc.6.3.3796.

Simonazzi, G., Miccoli, S., Salfi, N., Bonasoni, M. P., Bocciardi, R., Ravazzolo, R., Seri, M., Curti, A., Pilu, G., Rizzo, N., & Turchetti, D. (2012). A novel p63 mutation in a fetus with ultrasound detection of split hand/foot malformation. *Prenatal diagnosis*, *32*(3), 296–298. https://doi.org/10.1002/pd.2932.

Simpson, C. L., Kimble, D. C., Chandrasekharappa, S. C., NISC Comparative Sequencing Program, Alqosayer, K., Holzinger, E., Carrington, B., McElderry, J., Sood, R., Al-Souqi, G., Albacha-Hejazi, H., & Bailey-Wilson, J. E. (2023). A novel de novo TP63 mutation in whole-exome sequencing of a Syrian family with Oral cleft and ectrodactyly. *Molecular genetics & genomic medicine*, *11*(8), e2179. https://doi.org/10.1002/mgg3.2179.

Sowińska-Seidler, A., Socha, M., & Jamsheer, A. (2014). Split-hand/foot malformation - molecular cause and implications in genetic counseling. *Journal of applied genetics*, *55*(1), 105–115. https://doi.org/10.1007/s13353-013-0178-5.

van Bokhoven, H., Hamel, B. C., Bamshad, M., Sangiorgi, E., Gurrieri, F., Duijf, P. H., Vanmolkot, K. R., van Beusekom, E., van Beersum, S. E., Celli, J., Merkx, G. F., Tenconi, R., Fryns, J. P., Verloes, A., Newbury-Ecob, R. A., Raas-Rotschild, A., Majewski, F., Beemer, F. A., Janecke, A., Chitayat, D., … Brunner, H. G. (2001). p63 Gene mutations in eec syndrome, limb-mammary syndrome, and isolated split hand-split foot malformation suggest a genotype-phenotype correlation. *American journal of human genetics*, *69*(3), 481–492. https://doi.org/10.1086/323123.

van Bokhoven, H., & Brunner, H. G. (2002). Splitting p63. *American journal of human genetics*, *71*(1), 1–13. https://doi.org/10.1086/341450.

Wang, X., Yang, J., Tao, A. L., Yang, W. L., & Zhang, H. J. (2009). Mutation analysis of p63 gene in the first Chinese family with ADULT syndrome. *Chinese medical journal*, *122*(16), 1867–1871.

Wei, J., Xue, Y., Wu, L., Ma, J., Yi, X., Zhang, J., Lu, B., Li, C., Shi, D., Shi, S., Feng, X., & Cai, T. (2012). Analysis of large phenotypic variability of EEC and SHFM4 syndromes caused by K193E mutation of the TP63 gene. *PloS one*, *7*(5), e35337. https://doi.org/10.1371/journal.pone.0035337.

Whittington, A., Stein, S., & Kenner-Bell, B. (2016). Acro-Dermato-Ungual-Lacrimal-Tooth Syndrome: An Uncommon Member of the Ectodermal Dysplasias. *Pediatric dermatology*, *33*(5), e322–e326. https://doi.org/10.1111/pde.12938.

Xu, N., Shi, W., Cao, X., Zhou, X., Jin, L., Huang, H. F., Chen, S., & Xu, C. (2023). Parental mosaicism detection and preimplantation genetic testing in families with multiple transmissions of de novo mutations. *Journal of medical genetics*, *60*(9), 910–917. https://doi.org/10.1136/jmg-2022-108920.

Yamoto, K., Saitsu, H., Nishimura, G., Kosaki, R., Takayama, S., Haga, N., Tonoki, H., Okumura, A., Horii, E., Okamoto, N., Suzumura, H., Ikegawa, S., Kato, F., Fujisawa, Y., Nagata, E., Takada, S., Fukami, M., & Ogata, T. (2019). Comprehensive clinical and molecular studies in split-hand/foot malformation: identification of two plausible candidate genes (LRP6 and UBA2). *European journal of human genetics : EJHG*, *27*(12), 1845–1857. https://doi.org/10.1038/s41431-019-0473-7.

Yang, X., Lin, X., Zhu, Y., Luo, J., & Lin, G. (2018). Genetic analysis of a congenital split‑hand/split‑foot malformation 4 pedigree. *Molecular medicine reports*, *17*(6), 7553–7558. https://doi.org/10.3892/mmr.2018.8838.

Yang, Y., Huang, L. Y., Han, J., & Li, D. Z. (2017). Prenatal diagnosis of Ectrodactyly-Ectodermal dysplasia-Cleft (EEC) syndrome in a Chinese woman with a TP63 mutation. *European journal of obstetrics, gynecology, and reproductive biology*, *213*, 146–147. https://doi.org/10.1016/j.ejogrb.2017.04.012.

Yin, W., Ye, X., Shi, L., Wang, Q. K., Jin, H., Wang, P., & Bian, Z. (2010). TP63 gene mutations in Chinese P63 syndrome patients. *Journal of dental research*, *89*(8), 813–817. https://doi.org/10.1177/0022034510366804.

Yu, K., Dou, J., Huang, W., Wang, F., & Wu, Y. (2022). Expanding the genetic spectrum of tooth agenesis using whole-exome sequencing. *Clinical genetics*, *102*(6), 503–516. https://doi.org/10.1111/cge.14225.

Zhang, L., Pan, L., Teng, Y., Liang, D., Li, Z., & Wu, L. (2021). Molecular diagnosis for 55 fetuses with skeletal dysplasias by whole-exome sequencing: A retrospective cohort study. *Clinical genetics*, *100*(2), 219–226. https://doi.org/10.1111/cge.13976.

Zenteno, J. C., Berdón-Zapata, V., Kofman-Alfaro, S., & Mutchinick, O. M. (2005). Isolated ectrodactyly caused by a heterozygous missense mutation in the transactivation domain of TP63. American journal of medical genetics. Part A, 134A(1), 74–76. https://doi.org/10.1002/ajmg.a.30277.

Zheng, J., Liu, H., Zhan, Y., Liu, Y., Wong, S. W., Cai, T., Feng, H., & Han, D. (2019). Tooth defects of EEC and AEC syndrome caused by heterozygous TP63 mutations in three Chinese families and genotype-phenotype correlation analyses of TP63-related disorders. *Molecular genetics & genomic medicine*, *7*(6), e704. https://doi.org/10.1002/mgg3.704.

Zhou, X., Zhang, C., Fan, L., Wu, S., Yao, S., Wang, L., Zhong, W., Ma, L., & Pan, Y. (2023). A TP63 mutation identified in a Han Chinese family with ectodermal dysplasia. *Archives of oral biology*, *152*, 105731. https://doi.org/10.1016/j.archoralbio.2023.105731.

Zhuang, J., Li, Y., Chen, Y., Zhang, H., Liu, S., Hu, M., & Chen, C. (2025). Molecular characterization of a rare TP63 variant associated with split-hand/split-foot malformation 4 and incomplete penetrance: disruption of the p63-Dlx signaling pathway. BMC genomics, 26(1), 113. https://doi.org/10.1186/s12864-025-11297-3.
